# Alterations of cohesin complex genes in acute myeloid leukemia: differential co-mutations, clinical presentation and impact on outcome

**DOI:** 10.1038/s41408-023-00790-1

**Published:** 2023-01-24

**Authors:** Jan-Niklas Eckardt, Sebastian Stasik, Christoph Röllig, Tim Sauer, Sebastian Scholl, Andreas Hochhaus, Martina Crysandt, Tim H. Brümmendorf, Ralph Naumann, Björn Steffen, Volker Kunzmann, Hermann Einsele, Markus Schaich, Andreas Burchert, Andreas Neubauer, Kerstin Schäfer-Eckart, Christoph Schliemann, Stefan W. Krause, Regina Herbst, Mathias Hänel, Maher Hanoun, Ulrich Kaiser, Martin Kaufmann, Zdenek Rácil, Jiri Mayer, Tiago Cerqueira, Frank Kroschinsky, Wolfgang E. Berdel, Hubert Serve, Carsten Müller-Tidow, Uwe Platzbecker, Claudia D. Baldus, Johannes Schetelig, Timo Siepmann, Martin Bornhäuser, Jan Moritz Middeke, Christian Thiede

**Affiliations:** 1 Department of Internal Medicine I, University Hospital Carl Gustav Carus, Dresden, Germany; 2 Division of Health Care Sciences, Dresden International University, Dresden, Germany; 3 German Cancer Research Center (DKFZ) and Medical Clinic V, University Hospital Heidelberg, Heidelberg, Germany; 4 Department of Internal Medicine II, Jena University Hospital, Jena, Germany; 5 Department of Hematology, Oncology, Hemostaseology, and Cell Therapy, University Hospital RWTH Aachen, Aachen, Germany; 6 Medical Clinic III, St. Marien-Hospital Siegen, Siegen, Germany; 7 Medical Clinic II, University Hospital Frankfurt, Frankfurt (Main), Germany; 8 Medical Clinic and Policlinic II, University Hospital Würzburg, Würzburg, Germany; 9 Department of Hematology, Oncology and Palliative Care, Rems-Murr-Hospital Winnenden, Winnenden, Germany; 10 Department of Hematology, Oncology and Immunology, Philipps-University-Marburg, Marburg, Germany; 11 Department of Internal Medicine V, Paracelsus Medizinische Privatuniversität and University Hospital Nurnberg, Nurnberg, Germany; 12 Department of Medicine A, University Hospital Münster, Münster, Germany; 13 Medical Clinic V, University Hospital Erlangen, Erlangen, Germany; 14 Medical Clinic III, Chemnitz Hospital AG, Chemnitz, Germany; 15 Department of Hematology, University Hospital Essen, Essen, Germany; 16 Medical Clinic II, St. Bernward Hospital, Hildesheim, Germany; 17 Department of Hematology, Oncology and Palliative Care, Robert-Bosch-Hospital, Stuttgart, Germany; 18 Department of Internal Medicine, Hematology and Oncology, Masaryk University Hospital, Brno, Czech Republic; 19 Medical Clinic I Hematology and Celltherapy, University Hospital Leipzig, Leipzig, Germany; 20 Department of Internal Medicine, University Hospital Kiel, Kiel, Germany; 21 DKMS Clinical Trials Unit, Dresden, Germany; 22 Department of Neurology, University Hospital Carl Gustav Carus, Technische Universität Dresden, Dresden, Germany; 23 German Consortium for Translational Cancer Research DKTK, Heidelberg, Germany; 24 National Center for Tumor Disease (NCT), Dresden, Germany

**Keywords:** Acute myeloid leukaemia, Genetics research

## Abstract

Functional perturbations of the cohesin complex with subsequent changes in chromatin structure and replication are reported in a multitude of cancers including acute myeloid leukemia (AML). Mutations of its *STAG2* subunit may predict unfavorable risk as recognized by the 2022 European Leukemia Net recommendations, but the underlying evidence is limited by small sample sizes and conflicting observations regarding clinical outcomes, as well as scarce information on other cohesion complex subunits. We retrospectively analyzed data from a multi-center cohort of 1615 intensively treated AML patients and identified distinct co-mutational patters for mutations of *STAG2*, which were associated with normal karyotypes (NK) and concomitant mutations in *IDH2*, *RUNX1, BCOR, ASXL1*, and *SRSF2*. Mutated *RAD21* was associated with NK, mutated *EZH2, KRAS, CBL*, and *NPM1*. Patients harboring mutated *STAG2* were older and presented with decreased white blood cell, bone marrow and peripheral blood blast counts. Overall, neither mutated *STAG2, RAD21, SMC1A* nor *SMC3* displayed any significant, independent effect on clinical outcomes defined as complete remission, event-free, relapse-free or overall survival. However, we found almost complete mutual exclusivity of genetic alterations of individual cohesin subunits. This mutual exclusivity may be the basis for therapeutic strategies via synthetic lethality in cohesin mutated AML.

## Introduction

Acute myeloid leukemia (AML) is a genetically complex disease. The recently revised WHO classification acknowledges a variety of genetically defined alterations which constitute distinct disease entities [1]. Correspondingly, our understanding of myeloid neoplasms moves away from somewhat arbitrary numerical counts of bone marrow blasts and toward an appreciation of genetic drivers of disease as is acknowledged in the revised International Consensus Criteria [2]. On this basis, the recently revised European Leukemia Net (ELN) recommendations broaden the spectrum of clinically relevant genetic alterations with respect to individual patient risk warranting treatment that is adjusted to individual low-, intermediate-, and high-risk molecular alterations and cytogenetics [3]. In these updated definitions, mutations of the cohesin subunit SA-2 (*STAG2*) are recognized as a defining alteration of AML with myelodysplasia-related gene mutations (in absence of other defining alterations) irrespective of prior presence of myelodysplastic neoplasms [3]. Further, mutated *STAG2* is defined as a prognostic marker of high-risk (if not co-occurring with favorable risk AML subtypes) incentivizing intensive treatment and, potentially, allogeneic hematopoietic stem cell transplantation (HCT) [3].

STAG2, double-strand-break repair protein rad21 homologue (RAD21), and structural maintenance of chromosomes (SMC) proteins 1 A (SMC1A) and 3 (SMC3) form the four core units of the cohesin complex, a ring-like protein complex that encircles sister chromatids during replication and initiates metaphase-to-anaphase-transition upon sister chromatid release [4]. Additionally, the cohesion complex plays a key role in regulation of both structure and function of chromatin where it is recruited to chromatin binding sites via CCCTF-binding-factor [5]. Since the initial discovery of the cohesin complex in 1997(refs. [6, 7]), genetic alterations have been detected in a multitude of malignant neoplasms [8] including bladder cancer [9–11], Ewing sarcoma [12–14], endometrial cancer [15], glioblastoma [16], and myeloid malignancies [17–24].

While initially inactivating mutations of the cohesin complex were thought to promote carcinogenesis via aberrant segregation of sister chromatids and subsequent aneuploidy, especially recent findings of altered cohesin subunits in commonly euploid myeloid malignancies (with the exception of myeloid leukemias associated with Down Syndrome [18]) hint at more complex mechanisms of pathogenesis [4]. For instance, the finding that cohesin-CCCTF-binding-factor sites are frequently altered in cancer cells underlines the cohesins’ function in three-dimensional chromosome organization as a key component of carcinogenesis in a variety of neoplasms [25–27]. Further, inactivation of cohesin subunits may result in a complete collapse of topologically-associating-domain (TAD) structure [28–30]. Additionally, mutated cohesin subunits appear to play a role in stemness and differentiation in hematopoietic stem cells (HSC). Inactivation of *STAG2*, *RAD21*, *SMC1A*, and *SMC3* was found to promote stem cell self-renewal in human and mouse HSCs in vitro and subunit-specific knockout mice were found to bear changes in erythroid and myeloid differentiation mimicking myeloproliferative disorders similar to early human leukemogenesis [31–33]. This results in a proliferation advantage hinting at a key function of the cohesin complex in regulating cellular differentiation [31–33].

Taken together, these findings suggest a multi-facetted role of the cohesin complex and its individual subunits in human carcinogenesis. The impact of individually altered cohesin subunits on patient outcome in AML is unclear as previous studies have suggested unfavorable [17], favorable [19] as well as no prognostic impact [20]. Therefore, we aimed to identify distinct co-mutational patters for mutations of *STAG2* and other proteins of the cohesion complex that help to predict clinical outcomes in a large multicentric cohort of adult patients with AML.

## Methods

### Data set and definitions

We retrospectively analyzed a cohort of 1615 adult AML patients that were treated in previously reported multicenter trials (AML96(ref. [34]) [NCT00180115], AML2003(ref. [35]) [NCT00180102], AML60 + (ref. [36]) [NCT 00180167], and SORAML(ref. [37]) [NCT00893373]) or registered in the bio-registry of the German Study Alliance Leukemia (SAL [NCT03188874]) which encompasses 59 centers specialized in the treatment of hematologic neoplasms across Germany and the Czech Republic. Patients were eligible based on diagnosis of AML according to WHO criteria [1], age ≥ 18 years, curative treatment intent and available biomaterial at diagnosis. Prior to analysis, all patients gave their written informed consent according to the revised Declaration of Helsinki [38]. All studies were approved by the Institutional Review Board of the Technical University Dresden (EK 98032010). Complete remission (CR) and survival times including event-free (EFS), relapse-free (RFS), and overall survival (OS) were defined according to ELN2022 criteria [3]. Patients were retrospectively re-stratified into ELN2022 risk groups [3]. Since patients from earlier clinical trials were only re-stratified according to ELN2022 criteria, study accrual was not influence based on ELN risk. A summary of individual study protocols is provided in table S1. AML was defined as *de novo* when no prior malignancy and no prior treatment with chemo- and/or radiotherapy was reported. AML was defined as secondary (sAML) when prior myeloid neoplasms were reported, and therapy-associated (tAML) when prior exposure to chemo- and/or radiotherapy was reported.

### Molecular analysis and cytogenetic analysis

Pre-treatment peripheral blood or bone marrow aspirates were screened for genetic alterations using next-generation sequencing (NGS) with the TruSight Myeloid Sequencing Panel (Illumina, San Diego, CA, USA) covering 54 genes (table S2) that are associated with myeloid neoplasms including full coding exons for *SMC1A, RAD21*, and *STAG2* and relevant exons (10, 13, 19, 23, 25, and 28) for *SMC3* according to the manufacturer’s recommendations as previously reported [39, 40]. DNA was extracted using the DNeasy blood and tissue kit (Qiagen, Hilden, Germany) and quantified with the NanoDrop spectrophotometer. Pooled samples were sequenced paired-end (150 bp PE) on a NextSeq NGS-instrument (Illumina). Sequence data alignment of demultiplexed FastQ files, variant calling and filtering was performed with the Sequence Pilot software package (JSI medical systems GmbH, Ettenheim, Germany) with default settings and a 5% variant allele frequency (VAF) mutation calling cut-off. Human genome build HG19 was used as reference genome for mapping algorithms. Dichotomization of dominant and subclonal (or secondary) mutations was performed by comparing VAFs of detected mutations with VAFs of co-mutated driver variants. For resolution of putative subclonal mutations a minimum difference of 10% VAF was applied. For cytogenetic analysis, standard techniques for chromosome banding and fluorescence-in-situ-hybridization (FISH) were used.

### Statistical analysis

Statistical analysis was performed using STATA BE 17.0 (Stata Corp, College Station, TX, USA). All tests were carried out as two-sided tests. Statistical significance was determined using a significance level α of 0.05. Fisher’s exact test was used to compare categorical variables. Normality was assessed using the Shapiro-Wilk test. If the assumption of normality was met, continuous variables between two groups were analyzed using the two-sided unpaired t-test. If the assumption of normality was violated, continuous variables between two groups were analyzed using the Wilcoxon rank sum test. With regard to outcome variables, patients were analyzed on a complete case basis. Univariate analysis was carried out using logistic regression to obtain odds ratios (OR). Time-to-event analysis was performed using Cox-proportional hazard models to obtain hazard ratios (HR) as well as the Kaplan-Meier-method and the log-rank-test. For survival times, OR and HR, 95%-confidence-intervals (95%-CI) are reported. Multivariable models were adjusted for ELN2022 categories and age. In the case of AML with mutated *STAG2*, additional adjustments were performed in multivariable analysis for frequently co-mutated genes with an established impact on patient outcome according to ELN2022 definitions [3]. Median follow-up time was calculated using the reverse Kaplan-Meier method [41].

## Results

### Alterations of cohesin complex genes are recurrent events in AML with distinct clinical presentation

Alterations of any of genes of the cohesin complex (i.e. *STAG2, RAD21*, *SMC1A* and *SMC3*) were found in 184 of 1615 patients (11.4%). With the exception of one patient harboring both mutated *STAG2* and *SMC3*, alterations of cohesin complex genes were found to be mutually exclusive. With respect to clinical presentation, patients harboring mutations in genes of the cohesin complex had significantly lower white blood cell count (WBC, median 12.1*10^9^/l vs, 20.4*10^9^/l, *p* = 0.001), peripheral blood blast count (PBB, median 25.5% vs. 41.0%, *p* = 0.020), and bone marrow blast count (BMB, median 54.0% vs. 63.5%, *p* < 0.001) at initial diagnosis. Mutations in the cohesin complex were significantly associated with normal karyotypes (66.8% vs. 49.6%, *p* < 0.001), mutated *TET2* (25.0% vs. 18.5%, *p* = 0.046), *ASXL1* (17.9% vs. 7.1%, *p* < 0.001), and *SRSF2* (16.8% vs. 5.0%, *p* < 0.001), while inframe mutations in *CEBPA*-bZIP (7.6% vs. 9.1%, p = 0.008) and mutated *IKZF1* (0% vs. 3.2%, *p* = 0.007), *TP53* (2.7% vs. 7.7%, *p* = 0.009), complex karyotypes (6.0% vs. 12.4%, *p* = 0.022) and inv [16] or t(16;16) were rare (0.5% vs. 4.0%, *p* = 0.016) compared to cohesin wild-type AML. Table 1 shows baseline characteristics of patients with wild-type and cohesin mutated AML and Table S3 illustrates associations with other recurrent genetic alterations. Median follow-up time for the entire cohort was 89.5 months (95%-CI: 85.5-95.4).

**Table 1:** Baseline patient characteristics with respect to cohesin mutation status.

Parameter	Cohesin mutated	Cohesin wildtype	*p*
**n/N (%)**	184/1615 (11.4)	1426/1615 (88.3)	
**Age (years), median (IQR)**	57 (48.5-65.5)	53 (44.0-65.0)	0.112
**Sex, n (%)**			0.643
female	94 (49.7)	683 (47.9)	
male	95 (50.3)	743 (52.1)	
**Disease status, n (%)**			
de novo	141 (79.7)	1198 (84.9)	0.133
sAML	30 (16.9)	165 (11.7)	0.050
tAML	6 (3.4)	48 (3.4)	1.000
**extramedullary disease, n (%)**	24 (12.7)	190 (13.3)	1.000
**ELN-Risk 2022, n (%)**			
favorable	59 (31.2)	518 (36.3)	0.324
intermediate	32 (1518/16156.9)	392 (27.5)	**0.004**
adverse*	88 (46.6)*	498 (34.9)*	0.001*
missing	10 (5.3)	18 (1.3)	
**Complex karyotype, n (%)**			**0.022**
No	144 (92.9)	1123 (86.4)	
Yes	11 (7.1)	177 (13.6)	
**Normal karyotype, n (%)**			**<0.001**
No	46 (27.2)	627 (47.0)	
Yes	123 (72.8)	707 (53.0)	
**allogeneic HCT**			
in first CR	27 (14.7)	217 (15.2)	1.000
overall	55 (30.0)	464 (32.5)	0.613
missing	4 (2.2)	0	
**Laboratory, median (IQR)**			
WBC (10^9^/l)	12.1 (3.2-41.1)	20.4 (4.9-55.5)	**0.001**
HB (mmol/l)	6.0 (5.0-6.8)	5.9 (5.1-7.0)	0.764
PLT (10^9^/l)	49.0 (26.0-91.0)	50.5 (27.0-95.0)	0.899
LDH (U/l)	455.5 (281.0-824.0)	443.0 (771.0-281.0)	0.558
PBB (%)	25.5 (8.0-67.5)	41.0 (13.0-74.0)	**0.020**
BMB (%)	54.0 (40.0-72.5)	63.5 (45.0-79.5)	**<0.001**

*AML* acute myeloid leukemia, *sAML* secondary AML, *tAML* therapy-associated AML, *BMB* bone marrow blasts, *CR* complete remission, *HB* hemoglobin, *HCT* hematopoietic cell transplantation, *IQR* interquartile range, *n/N* number, *PBB* peripheral blood blasts, *PLT* platelet count, *WBC* white blood cell count. Boldface indicates statistical significance (*p* < 0.05). *It has to be taken into account that mutated *STAG2* is included as a marker of adverse risk in the novel ELN2022 risk stratification model and thus, partial collinearity may have diluted this specific result.

### AML with mutated *STAG2* shows a distinct co-mutational pattern and clinical presentation while patient outcome is not affected

**Fig. 1:**
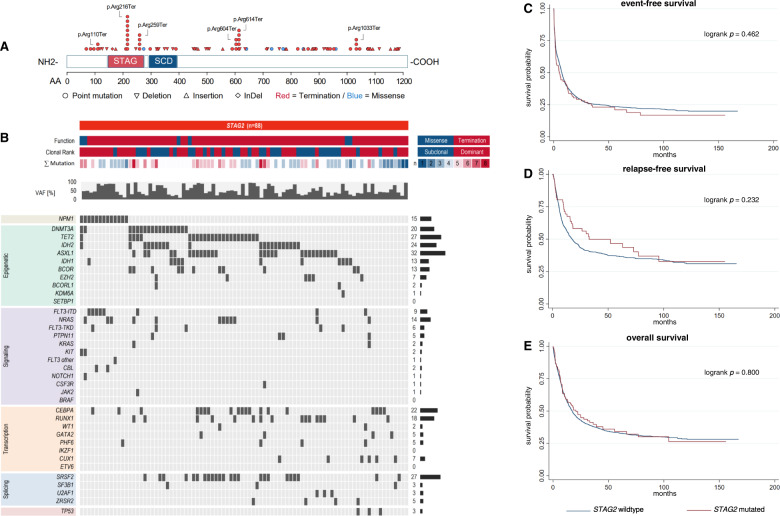
Distribution, co-mutational spectrum and survival analysis for AML with mutated *STAG2*. Graphic representation of the domain structure of *STAG2* and positions of *STAG2* mutations in 88 AML patients (**A**). Mutations of *STAG2* are categorized by function (missense=blue, termination=red), clonal rank (dominant=red/subclonal=blue), number of mutations, and variant allele fraction, and associated co-mutations (**B**). For detailed information on co-mutations and results of individual significance tests, see Tab. S4. Survival analysis using the Kaplan-Meier method and log-rank test for event-free (**C**), relapse-free (**D**) and overall survival (**E**) differentiating between *STAG2*-mutated and *STAG2*-wildtype AML.

Mutations in *STAG2* were the most frequent alterations of cohesin complex genes in the cohort (*n* = 88, 5.4%). The majority of *STAG2* mutations were nonsense mutations (*n* = 82, 93.2%), while missense mutations were rare (*n* = 6, 6.8%, Fig. 1A). Alterations of *STAG2* were more often dominant (58.0%) rather than subclonal (42.0%). At initial presentation, AML patients harboring mutated *STAG2* were significantly older (58 years vs. 55 years, *p* = 0.023) and had lower WBC (median 5.4 × 10^9^/l vs. 20.7 × 10^9^/l, *p* < 0.001), PBB (20.0% vs. 41.0%, *p* < 0.001), BMB (48.3% vs. 63.5%, *p* < 0.001) and LDH (median 342 U/l vs. 450.8 U/l, *p* = 0.001). Table S4 provides an overview of baseline characteristics of patients with *STAG2*-mutated AML. Alterations of *STAG2* were frequently associated with normal karyotypes (75.0% vs. 50.4%, *p* < 0.001), mutated *IDH2* (23.9% vs. 13.5%, *p* = 0.011), *RUNX1* (18.2% vs. 8.8%, *p* = 0.007), *BCOR* (12.5% vs. 4.3%, *p* = 0.002), *ASXL1* (31.8% vs. 6.9%, *p* < 0.001), *SRSF2* (27.3% vs. 5.1%, *p* < 0.001), *CUX1* (6.8% vs. 2.4%, *p* = 0.004), and *ZRSR2* (4.5% vs. 1.4%, *p* = 0.044). Compared to *STAG2*-wild-type AML, in-frame mutations of *CEBPA*-bZIP (5.7% vs. 9.1%, *p* = 0.004) as well as mutated *NPM1* (14.8% vs. 32.0%, *p* < 0.001) and *FLT3*-ITD (9.1% vs. 22.3%, *p* = 0.002) were significantly less common (Fig. 1B, Table S5). With respect to patient outcome, we found no difference in CR rate between patients with mutated or wild-type *STAG2* (63.6% vs. 66.8%, OR = 0.71, *p* = 0.138). EFS, RFS and OS did not differ (Table 2, Fig. 1C–E). Clonality, i. e. whether mutated *STAG2* was present in the dominant clone or detected at a subclonal level, did also not influence patient outcome. Mutations of *BCOR*, *RUNX1* and *ASXL1* are established markers of adverse risk and mutated *STAG2* has been added as an adverse marker in the recent ELN2022 recommendations [3]. Since patients that harbored mutations of *STAG2* showed a co-mutational pattern with significantly increased rates of co-mutated *BCOR*, *RUNX1* and *ASXL1*, we evaluated their individual influence on outcome in multivariable models. With respect to achievement of CR, mutated *STAG2* remained of no independent prognostic impact in a multivariable model adjusted for mutational status of *BCOR*, *RUNX1*, and *ASXL1* (Table S6) while mutated *RUNX1* and *ASXL1* showed significantly decreased ORs to achieve CR (*p* < 0.001 for both) in this model. Regarding survival times, multivariable models adjusted for mutations of *BCOR*, *RUNX1*, and *ASXL1* also showed no independent impact of mutated *STAG2* on EFS (Table S7), RFS (Table S8), and OS (Table S9). Contrastingly, mutated *RUNX1* showed significantly increased HRs for EFS, RFS, OS, while mutated *ASXL1* showed significantly increased HRs for EFS and OS, and mutated *BCOR* showed a significantly increased HR only for EFS in these multivariable models (Tables S6–8).

**Table 2:** Summary of outcomes for AML patients with mutations of cohesin subunit genes.

Outcome	mut. *STAG2*	wt*STAG2*	OR/HR	*p*
n/N (%)	88/1615 (5.5)	1527/1615 (94.5)		
CR rate, n (%)	56/88 (63.6%)	1079/1615 (66.8)	0.71 [0.45-1.11]	0.138
EFS	4.9 [1.9-11.9]	7.4 [6.6-8.1]	1.09 [0.86-1.39]	0.464
RFS	32.9 [15.8-95.6]	17.5 [14.8-20.6]	0.81 [0.57-1.15]	0.233
OS	20.7 [11.0-36.2]	17.3 [15.6-19.1]	0.97 [0.74-1.26]	0.800
	**mut**. ***RAD21***	**wt*RAD21***	**OR/HR**	***p***
n/N (%)	51/1615 (3.2)	1564/1615 (96.8)		
CR rate, n (%)	41/51 (80.4)	1094/1615 (67.7)	1.73 [0.86-3.48]	0.126
EFS	11.4 [5.4-41.4]	7.1 [6.4-7.9]	0.74 [0.53-1.03]	0.077
RFS	23.5 [8.3-108.3]	18.1 [15.5-21.2]	0.91 [0.61-1.36]	0.646
OS	20.6 [8.2-112.2]	17.5 [15.7-19.1]	0.85 [0.60-1.22]	0.386
	**mut**. ***SMC1A***	**wt*SMC1A***	**OR/HR**	***p***
n/N (%)	25/1615 (1.5)	1590/1615 (98.5)		
CR rate, n (%)	17/25 (68.0)	1118/1615 (69.2)	1.18 [0.46-3.00]	0.731
EFS	14.6 [9.3-n.r.]	7.1 [6.4-7.9]	0.61 [0.35-1.05]	0.075
RFS	n.r.	18.1 [15.5-21.4]	0.61 [0.29-1.28]	0.190
OS	11.4 [6.4-n.r.]	17.5 [15.7-19.2]	0.85 [0.49-1.47]	0.567
	**mut**. ***SMC3***	**wt*SMC3***	**OR/HR**	***p***
n/N (%)	20/1615 (1.2)	1595/1615 (98.8)		
CR rate, n (%)	12/20 (60.0)	1123/1615 (69.5)	0.83 [0.31-2.22]	0.708
EFS	9.8 [0.9-27.5]	7.1 [6.5-7.9]	0.94 [0.55-1.59]	0.809
RFS	25.8 [7.7-n.r.]	18.1 [15.5-21.4]	0.72 [0.34-1.52]	0.389
OS	21.4 [4.7-n.r.]	17.5 [15.7-19.1]	0.91 [0.51-1.60]	0.740

Survival times are displayed in months. Square brackets show 95%-confidence intervals.*CR* complete remission, *EFS* event-free survival, *HR* hazard ratio, *mut.* mutated, *n/N* number, *n.r.* not reached, *OR* odds ratio, *OS* overall survival, *RFS* relapse-free-survival, *wt* wild-type.

### AML with mutated *RAD21* shows a distinct co-mutational pattern while *RAD21* mutational status does not influence outcome

**Fig. 2:**
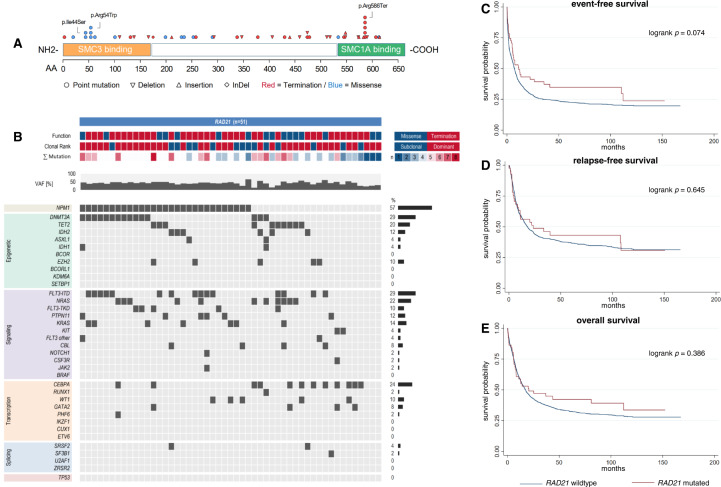
Distribution, co-mutational spectrum and survival analysis for AML with mutated *RAD21*. Graphic representation of the domain structure of *RAD21* and positions of *RAD21* mutations in 51 AML patients (**A**). Mutations of *RAD21* are categorized by function (missense=blue, termination=red), clonal rank (dominant=red/subclonal=blue), number of mutations, and variant allele fraction, and associated co-mutations (**B**). For detailed information on co-mutations and results of individual significance tests, see Tab. S10. Survival analysis using the Kaplan-Meier method and logrank test for event-free (**C**), relapse-free (**D**) and overall survival (**E**) differentiating between *RAD21* -mutated and *RAD21* -wildtype AML.

The second most common alteration was *RAD21* which was detected in 51 patients (3.2%), again mostly being nonsense (n = 35, 68.6%) rather than missense mutations (n = 16, 31.4%, Fig. 2A). Further, alterations of *RAD21* were mostly dominant (n = 36, 70.6%). With respect to baseline patient characteristics, patients harboring *RAD21* mutations showed significantly increased LDH (median 705.0 U/l vs. 440.2 U/l, *p* < 0.001) upon initial diagnosis. Table S10 provides an overview of baseline patient characteristics. Patients with mutated *RAD21* were most frequently categorized within the ELN2022 favorable risk group (51.0% vs. 35.2%, *p* = 0.037), while categorization in the ELN2022 adverse risk group was less prevalent (15.7% vs. 37.0%, *p* = 0.001). Concordantly, patients with mutated *RAD21* commonly had normal karyotypes (70.6% vs. 50.8%, *p* = 0.009), while complex aberrant karyotypes were rare (2.0 vs. 12.0%, *p* = 0.009). Common co-mutations compared to patients with wild-type *RAD21* were alterations in *EZH2* (9.8% vs. 3.7%, p = 0.046), *KRAS* (13.7% vs. 5.0%, p = 0.016), *CBL* (7.8% vs. 1.8%, p = 0.017), and *NPM1* (56.9% vs. 30.4%, p < 0.001, Fig. 2B). Mutated *RAD21* was mutually exclusive with mutated *SMC3*, *SMC1A* and *STAG2* as well as *TP53* (*p* = 0.047). Table S11 shows co-mutations of mutated *RAD21* in detail. With respect to patient outcome, we found no differences in CR rate, EFS, RFS and OS for patients with mutated vs. wild-type *RAD21* in general (Table 2, Fig. 2C-E). Clonality of mutated *RAD21* (dominant clone vs. subclonal) did not affect CR rate, EFS, RFS or OS.

### AML with either mutated *SMC3* or *SMC1A* does not differ from *SMC3*- or *SMC1A*-wild-type AML regarding clinical presentation, co-mutations, and outcome

**Fig. 3:**
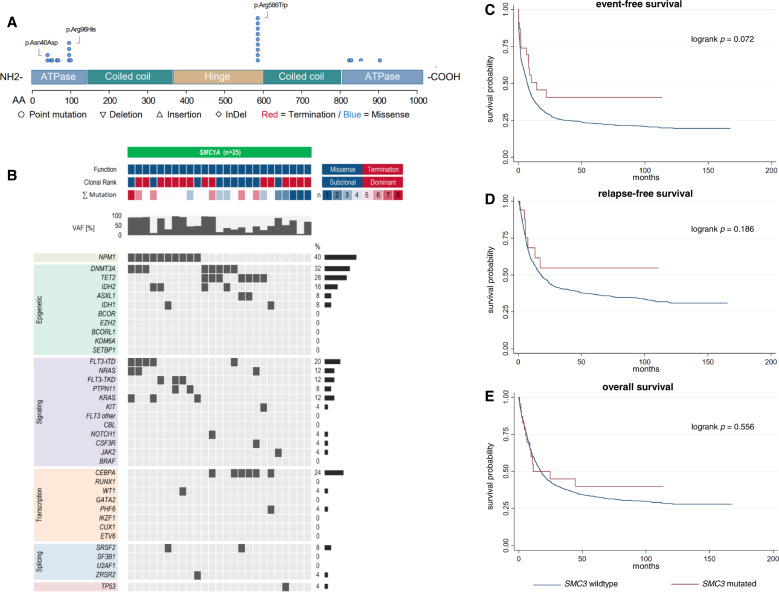
Distribution, co-mutational spectrum and survival analysis for AML with mutated *SMC1A*. Graphic representation of the domain structure of *SMC1A* and positions of *SMC1A* mutations in 25 AML patients (**A**). Mutations of *SMC1A* are categorized by function (missense=blue, termination=red), clonal rank (dominant=red/subclonal=blue), number of mutations, and variant allele fraction, and associated co-mutations (**B**). For detailed information on co-mutations and results of individual significance tests, see Tab. S13. Survival analysis using the Kaplan-Meier method and logrank test for event-free (**C**), relapse-free (**D**) and overall survival (**E**) differentiating between *SMC1A* -mutated and *SMC1A* -wildtype AML.

**Fig. 4:**
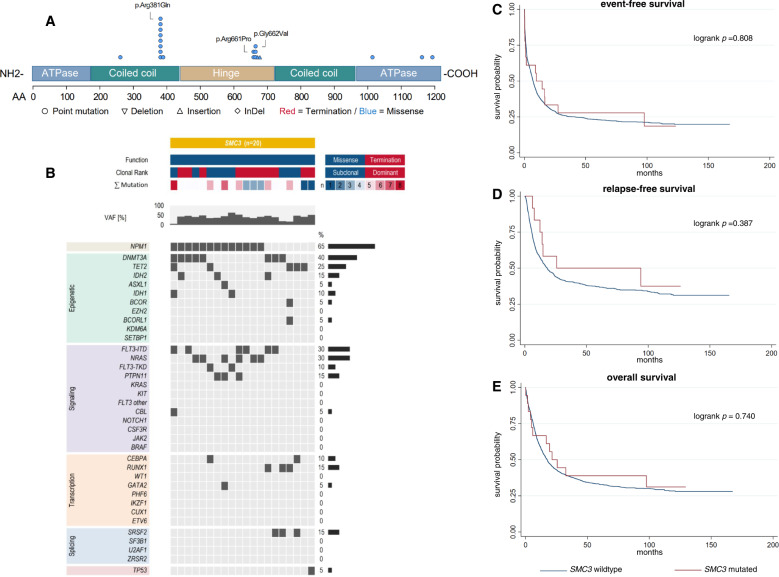
Distribution, co-mutational spectrum and survival analysis for AML with mutated *SMC3*. Graphic representation of the domain structure of *SMC3* and positions of *SMC3* mutations in 20 AML patients (**A**). Mutations of *SMC3* are categorized by function (missense=blue, termination=red), clonal rank (dominant=red/subclonal=blue), number of mutations, and variant allele fraction, and associated co-mutations (**B**). For detailed information on co-mutations and results of individual significance tests, see Tab. S14. Survival analysis using the Kaplan-Meier method and logrank test for event-free (**C**), relapse-free (**D**) and overall survival (**E**) differentiating between *SMC3*-mutated and *SMC3*-wildtype AML.

Twenty-five patients (1.5%) harbored alterations in *SMC1A*, while mutated *SMC3* was found in 20 patients (1.2%). Alterations in both *SMC1A* and *SMC3* were only detected as missense mutations (Fig. 3A, Fig. 4A) and the majority was found in the dominant clone (*SMC1A*: 60.0%, *SMC3*: 55.0%). There were neither differences in baseline clinical characteristics between patients with *SMC1A*-mutated vs. *SMC1A*-wild-type AML (Table S12) nor between patients with *SMC3*-mutated vs. *SMC3*-wild-type AML (Table S13). With respect to co-mutations, patients harboring mutated *SMC1A* showed significantly increased rates of t(8;21) (20.0% vs. 3.5%, *p* = 0.002) while no other associations were found (Fig. 3B, Table S14). Patients harboring *SMC3* mutations showed significantly increased co-mutations of *NPM1* (65.0% vs. 30.8%, *p* = 0.003) while no difference between mutated or wild-type *SMC3* was found for other alterations (Fig. 4B, Table S15). CR rate did not differ neither for patients with *SMC1A* mutations nor patients with *SMC3* mutations when compared to wildtype patients. With regard to survival times, again no difference was found both for patients with *SMC1A*-mutated vs. *SMC1A*-wildtype AML (Fig. 3C-E) as well as patients with *SMC3* mutations when compared to their wildtype counterparts (Fig. 4C-E). Further analysis with respect to clonality (dominant vs. subclonal) of the specific mutations did not show any differences for CR rates, EFS, RFS, or OS both for AML with mutated *SMC1A* and *SMC3*.

## Discussion

In our retrospective multi-center cohort study in 1615 intensively treated AML patients, we were able to ascertain distinct patterns of changes in cohesin complex genes, identify their association with other recurrent genetic alterations and clinical presentation as well as confirm their mutual exclusivity which may serve as a target for therapeutic approaches. We confirm that mutations in the genes of the cohesin complex are recurrent genetic events in AML with a reported frequency between 5.9-13.0%(ref. [15, 19, 20, 23, 24, 42]) which is in line with our cohort where 11.4% of patients harbored an alteration of cohesin complex genes. In accordance with previous studies(ref. [15, 19, 20, 23, 24, 42]), we found these mutations to be mutually exclusive with the exception of one patient bearing both mutated *STAG2* and *SMC3*. The inactivation of more than one subunit of the cohesin complex may result in structural collapse of topologically associated domains which may explain the mutual exclusivity of these gene alterations [4]. *STAG2* may form the exception as it has a functional homologue in *STAG1* that can potentially compensate for its malfunction in three-dimensional genome organization [29]. In the early days of research into the role of the cohesin complex in carcinogenesis, it has been hypothesized that its malfunction may lead to aberrant segregation of sister chromatids and consequently aneuploidy as a major driver in neoplastic transformation [8]. However, especially studies investigating mutations of the cohesin complex in myeloid neoplasms have refuted this claim since these alterations are commonly found in AML with euploid karyotypes [20, 24, 42]. Correspondingly, the rate of patients with normal karyotypes in our cohort was significantly increased while the rate of complex aberrant karyotypes was significantly decreased for patients with cohesin-mutated AML. These findings suggest alternate contributions of cohesin in carcinogenesis rather than mere aneuploidy and chromosomal instability. Alterations of cohesin subunit genes have both been described as early and late events in leukemogenesis [17, 20, 24, 31, 32, 43] suggesting a passenger rather than a driver function. However, in our cohort, the majority of cohesion complex mutations were detected in dominant clonal constellations, pointing at a potential role as an early event during AML initiation. Likewise, cohesin plays an important role in regulating the stemness and pluripotency of stem cells [31–33]. Thus, an interplay of alterations of cohesin genes with other genetic events in driver genes such as *NPM1* likely promotes malignant transformation. Mutated *NPM1* has been associated with alterations of cohesin complex genes [19, 20]. An interaction of cohesin proteins with NPM1 could be mediated by CCCTC-binding factor—a transcription factor that regulates tumor suppressor loci—which has been shown to bind and interact with both [44, 45], potentially contributing to their role in stem cell self-renewal [46, 47]. In our cohort, we found mutated *SMC3* and *RAD21* to be associated with mutated *NPM1* while *NPM1* mutations were less frequently associated with mutated *STAG2*. In comparison to their wildtype counterparts, patients with mutated *STAG2* more frequently also had mutated *IDH2, TET2, BCOR, ASXL1, SRSF2*, and *ZRSR2*. Further, patients with alterations of *RAD21* showed increased rates of co-occurring mutations in *EZH2*, *KRAS,* and *CBL* besides *NPM1*. An association of cohesin mutations with mutated *TET2*, *ASXL1, BCOR,* and *EZH2* has previously been reported [24, 42], however, it is important to note that different subunits of the cohesin complex show different co-mutational patterns.

The prognostic impact of cohesin mutations in AML has been unclear as studies are not only limited but also report conflicting results. Tsai et al. [19] report increased OS and disease-free survival for patients with cohesin mutations, which was confirmed by multivariable analysis in a cohort of 391 patients with de novo AML. In contrast, Thol et al. [20] found no impact of cohesin mutations on CR rate, RFS, and OS in a cohort of 389 intensively treated AML patients. In MDS, Thota et al. [24] reported decreased OS for patients with cohesin mutations, especially in *STAG2*-mutated MDS for patients who survived beyond 12 months. Commonly, previous studies were limited in sample size, often ranging between 300 and 600 patients. In our comparatively large cohort of 1615 intensively treated AML patients, we did not find a significant impact of any gene alterations of cohesin subunits on CR rate, EFS, RFS, or OS. The recently revised ELN2022 recommendations [3] introduce mutated *STAG2* as a prognostic marker of adverse risk (if no markers of favorable risk are co-occuring). Multivariable models adjusted for mutation status of *BCOR*, *ASXL1*, and *RUNX1*, which were more prevalent in *STAG2*-mutated AML patients, demonstrated no independent impact of mutated *STAG2* on patient outcome while these co-mutations had varying individual prognostic impact. Several reports agree that *STAG2* mutations are associated with sAML, and thus, as a part of corresponding compound attributes they are associated with the overall adverse impact of sAML on outcome [17, 43]. However, these mutations contribute only a minor part of this compound attributes. According to our observations such an adverse effect on outcome cannot be verified for the presence of STAG2 mutations per se. While the cohesin complex undoubtably plays a role in leukemogenesis, given the ambiguity of existing reports on cohesin’s (and *STAG2*’s) role in AML prognostication [19, 20, 24] caution may be warranted with respect to determining patient risk and ultimately treatment allocation. Nevertheless, it should be acknowledged that our study is limited by the fact that results are only available for intensively treated patients. The extent to which the reported results are also transferable to patients which receive less intensive regimens or targeted therapy remains to be evaluated.

While the prognostic impact of cohesin alterations in AML remains elusive, their co-mutational pattern with respect to mutual exclusivity may make them a viable option for targeted therapy. Mutually exclusive gene alterations may be utilized therapeutically via synthetic lethality [48]. If the alteration of one mutated gene provides a cancerous cell with a survival advantage as long as a second gene remains unaltered, the alteration or inhibition of the second gene or its gene product may confer apoptosis specifically in cells carrying the initial alteration [49, 50]. Synthetic lethality via inhibition of mediators of replication fork stability such as poly ADP-ribose polymerase (PARP) has been demonstrated in *BRCA*-mutated breast, ovarian, pancreatic, and prostate cancer [51]. The functional homologues *STAG1* and *2* offer the possibility for a synthetically lethal therapeutic strategy via PARP inhibition. In glioblastoma cells, Bailey et al. [52] have demonstrated that mutated *STAG2* significantly increases the sensitivity to PARP inhibition. Further, Black et al. [53] found *STAG2*-deficient leukemic cells to bear a significantly higher susceptibility to treatment with talazoparib. Currently, a phase 1 study is ongoing investigating the safety and efficacy of talazoparib for cohesin-mutated AML and MDS with excessive blasts (NCT03974217) [54].

In summary, we report distinct co-mutational and clinical patterns for mutated *STAG2* and *RAD21* in a large sample of AML patients while mutated *SMC3* and *SMC1A* lacked such patterns. However, no cohesin subunit—including mutated STAG2 that was recently added to the ELN2022 criteria as a marker of adverse risk—showed any impact on patient outcome regarding the achievement of CR, EFS, RFS, or OS. While we did not find a prognostic impact of cohesin alterations in AML, their mutual exclusivity may make them a potential target for therapeutic approaches based on synthetic lethality.

## Supplementary information

supplemental tables

## Data Availability

The datasets generated during and analyzed during the current study are available in the Kaggle repository, 10.34740/KAGGLE/DSV/4816451.
