# Supplementary material for: Alterations of cohesin complex genes in acute myeloid leukemia: differential co-mutations, clinical presentation and impact on outcome
Source: Blood Cancer J. 2023 Jan 24;13(1):18. doi: 10.1038/s41408-023-00790-1 (PMC9873811; doi:10.1038/s41408-023-00790-1)
Supplement: Supplementary file 1 — supplemental tables [file 41408_2023_790_MOESM1_ESM.docx]

## **Supplements**

| **trial name** | **clinicaltrials.gov identifier** | **trial duration** | **protocol summary** |
| --- | --- | --- | --- |
| AML96 | NCT00180115 | 1996-2008 | risk-adapted postremission treatment regarding allogeneic stem cell transplantation for high-risk AML and related allogeneic and autologous stem cell transplantation for standard-risk AML, and randomization between intermediate-dose and high-dose cytarabine within the first post-remission course |
| AML2003 | NCT00180102 | 2003-2009 | early allogeneic stem cell transplantation in post-induction aplasia for high-risk AML, factorial design with four therapy arms with two factors of two stages (intensified vs. standard therapy and cytarabine vs. cytarabine + mitoxantrone + amsacrin) |
| AML60+ | NCT00180167 | 2005-2010 | Patients ≥ 60 years, mitoxantron on day 1,2,3 + cytarabine on days 1,3,5,7 vs. DA 7+3 |
| SORAML | NCT00893373 | 2011-2014 | Standard therapy + sorafenib vs. standard therapy + placebo |
| SAL bioregistry | NCT03188874 | 2010-present | Prospective registry of AML patients |

**Table S1.** Summary of clinical trial protocols and treatment regimens for previous clinical trials patient data was retrieved from for the purpose of retrospective analyses.

| TruSight Myeloid Sequencing Panel | | | | |
| --- | --- | --- | --- | --- |
| *ABL1* | *CEBPA* | *HRAS* | *MYD88* | *SF3B1* |
| *ASXL1* | *CSF3R* | *IDH1* | *NOTCH1* | *SMC1A* |
| *ATRX* | *CUX1* | *IDH2* | *NPM1* | *SMC3* |
| *BCOR* | *DNMT3A* | *IKZF1* | *NRAS* | *SRSF2* |
| *BCORL1* | *ETV6/TEL* | *JAK2* | *PDGFRA* | *STAG2* |
| *BRAF* | *EZH2* | *JAK3* | *PHF6* | *TET2* |
| *CALR* | *FBXW7* | *KDM6A* | *PTEN* | *TP53* |
| *CBL* | *FLT3* | *KIT* | *PTPN11* | *U2AF1* |
| *CBLB* | *GATA1* | *KRAS* | *RAD21* | *WT1* |
| *CBLC* | *GATA2* | *MLL* | *RUNX1* | *ZRSR2* |
| *CDKN2A* | *GNAS* | *MPL* | *SETBP1* |  |

**Table S2 Myeloid Gene Panel.** Summary of the 54 genes targeted by the TruSight Myeloid Sequencing Panel (Illumina, San Diego, CA, USA).

|  |  | **Cohesin mut. (abs)** | **Cohesin mut. (rel) %** | **Cohesin wt. (abs)** | **Cohesin wt. (rel) %** | ***p*** |
| --- | --- | --- | --- | --- | --- | --- |
|  | N=1615 | 184 | 11.4 | 1426 | 88.3 |  |
| Epigenetic | DNMT3A | 49 | 26.6 | 407 | 28.5 | 0.664 |
|  | IDH1 | 17 | 9.2 | 132 | 9.3 | 1.000 |
|  | IDH2 | 34 | 18.5 | 194 | 13.6 | 0.091 |
|  | TET2 | 46 | 25.0 | 264 | 18.5 | **0.046** |
|  | BCOR | 12 | 6.5 | 64 | 4.5 | 0.264 |
|  | BCORL1 | 3 | 1.6 | 57 | 4.0 | 0.145 |
|  | ASXL1 | 33 | 17.9 | 101 | 7.1 | **<0.001** |
|  | EZH2 | 11 | 6.0 | 52 | 3.6 | 0.153 |
| Transcription | CEBPA | 39 | 21.2 | 219 | 15.4 | 0.091 |
|  | CEBPA, biallelic | 11 | 6.0 | 81 | 5.7 | 0.731 |
|  | CEBPA-TAD | 8 | 4.3 | 29 | 2.0 | 0.062 |
|  | CEBPA-bZIP (in frame) | 14 | 7.6 | 130 | 9.1 | **0.008** |
|  | CUX1 | 6 | 3.3 | 36 | 2.5 | 0.469 |
|  | GATA2 | 9 | 4.9 | 88 | 6.2 | 0.621 |
|  | IKZF1 | 0 | 0.0 | 45 | 3.2 | **0.007** |
|  | PHF6 | 6 | 3.3 | 47 | 3.3 | 1.000 |
|  | RUNX1 | 20 | 10.9 | 131 | 9.2 | 0.501 |
|  | WT1 | 8 | 4.3 | 110 | 7.7 | 0.131 |
|  | ETV6 | 0 | 0.0 | 15 | 1.1 | 0.402 |
| Signaling | FLT3-ITD | 34 | 18.5 | 314 | 22.0 | 0.256 |
|  | FLT3-TKD | 15 | 8.2 | 54 | 3.8 | 0.236 |
|  | NRAS | 32 | 17.4 | 218 | 15.3 | 0.450 |
|  | KRAS | 12 | 6.5 | 73 | 5.1 | 0.385 |
|  | KIT | 5 | 2.7 | 74 | 5.2 | 0.201 |
|  | NOTCH1 | 3 | 1.6 | 29 | 2.0 | 1.000 |
|  | CSF3R | 3 | 1.6 | 26 | 1.8 | 1.000 |
|  | CBL | 7 | 3.8 | 25 | 1.8 | 0.084 |
|  | PTPN11 | 15 | 8.2 | 100 | 7.0 | 0.544 |
| Splicing | SF3B1 | 4 | 2.2 | 42 | 2.9 | 0.813 |
|  | SRSF2 | 31 | 16.8 | 71 | 5.0 | **<0.001** |
|  | U2AF1 | 3 | 1.6 | 42 | 2.9 | 0.473 |
|  | ZRSR2 | 5 | 2.7 | 20 | 1.4 | 0.195 |
| other | TP53 | 5 | 2.7 | 110 | 7.7 | **0.009** |
|  | NPM1 | 65 | 35.3 | 439 | 30.8 | 0.313 |
| cytogenetics | normal karyotype | 123 | 66.8 | 707 | 49.6 | **<0.001** |
|  | complex karyotype | 11 | 6.0 | 177 | 12.4 | **0.022** |
|  | t(8;21)(q22;q22.1) | 10 | 5.4 | 51 | 3.6 | 0.134 |
|  | inv(16)(p13.1q22) or t(16;16)(p13.1;q22) | 1 | 0.5 | 57 | 4.0 | **0.016** |

**Table S3 Co-mutational pattern of AML with mutations in genes of the cohesin complex.** Abbreviations: absolute (abs.), number (n/N), relative (rel.). Bold typing indicates statistical significance (*p*<0.05).

| **Parameter** | ***STAG2* mutated** | ***STAG2* wildtype** | ***p*** |
| --- | --- | --- | --- |
| **n/N (%)** | 88/1615 (5.5) | 1527/1615 (94.5) |  |
| **Age (years), median (IQR)** | 58 (49.0-67.5) | 55.0 (44.0-65.0) | **0.023** |
| **Sex, n (%)** |  |  | 1.000 |
| female | 43 (48.9) | 728 (48.0) |  |
| male | 45 (51.1) | 790 (52.0) |  |
| **Disease status, n (%)** |  |  |  |
| de novo | 61 (70.9) | 1278 (85.1) | 0.002 |
| sAML | 22 (25.6) | 173 (11.5) | **<0.001** |
| tAML | 3 (3.5) | 51 (3.4) | 0.764 |
| **extramedullary disease, n (%)** | 8 (9.1) | 206 (13.5) | 0.258 |
| **ELN-Risk 2022, n (%)** |  |  |  |
| favorable* | 12 (13.6)* | 565 (37.0)* | **<0.001*** |
| intermediate* | 6 (6.8)* | 418 (27.4)* | **<0.001*** |
| adverse* | 70 (79.6)* | 516 (33.8)* | **<0.001*** |
| missing | 0 | 28 (1.8) |  |
| **Complex karyotype, n (%)** |  |  | 0.107 |
| No | 63 (92.6) | 1204 (86.8) |  |
| Yes | 5 (7.4) | 183 (13.2) |  |
| **Normal karyotype, n (%)** |  |  | **<0.001** |
| No | 19 (23.8) | 654 (46.0) |  |
| Yes | 61 (76.2) | 769 (54.0) |  |
| **allogeneic HCT** |  |  |  |
| in first CR | 18 (20.5) | 226 (14.0) | 0.168 |
| overall | 37 (42.0) | 482 (29.9) | **0.047** |
| **Laboratory, median (IQR)** |  |  |  |
| WBC (10^9^/l) | 5.4 (2.3-16.6) | 20.7 (4.9-55.9) | **<0.001** |
| HB (mmol/l) | 6.1 (5.0-7.7) | 5.9 (5.0-7.0) | 0.424 |
| PLT (10^9^/l) | 50.5 (25.0-104.0) | 50.0 (28.0-94.0) | 0.852 |
| LDH (U/l) | 342.0 (228.0-547.0) | 450.8 (285.0-787.0) | **0.001** |
| PBB (%) | 20.0 (4.0-52.0) | 41.0 (13.0-74.0) | **<0.001** |
| BMB (%) | 48.3 (32.0-65.0) | 63.5 (45.5-79.5) | **<0.001** |

**Table S4 Baseline patient characteristics with respect to *STAG2* mutation status.**Abbreviations: acute myeloid leukemia (AML), secondary AML (sAML), therapy-associated AML (tAML), bone marrow blasts (BMB), complete remission (CR), hemoglobin (HB), hematopoietic cell transplantation (HCT), interquartile range (IQR), number (n/N), peripheral blood blasts (PBB), platelet count (PLT), white blood cell count (WBC). Boldface indicates statistical significance (*p*<0.05). *It has to be taken into account that mutated *STAG2* is included as a marker of adverse risk in the novel ELN2022 risk stratification model and thus, partial collinearity may have diluted this specific result.

|  |  | ***STAG2* mut. (abs)** | ***STAG2* mut. (rel) %** | ***STAG2* wt. (abs.)** | ***STAG2* wt (rel) %** | ***p*** |
| --- | --- | --- | --- | --- | --- | --- |
|  | N=1615 | 88 | 5.4 | 1527 | 94.0 |  |
| Epigenetic | *DNMT3A* | 18 | 20.5 | 437 | 28.6 | 0.113 |
|  | *IDH1* | 11 | 12.5 | 137 | 9.0 | 0.257 |
|  | *IDH2* | 21 | 23.9 | 206 | 13.5 | **0.011** |
|  | *TET2* | 24 | 27.3 | 285 | 18.7 | 0.069 |
|  | *BCOR* | 11 | 12.5 | 65 | 4.3 | **0.002** |
|  | *BCORL1* | 2 | 2.3 | 58 | 3.8 | 0.769 |
|  | *ASXL1* | 28 | 31.8 | 105 | 6.9 | **<0.001** |
|  | *EZH2* | 6 | 6.8 | 57 | 3.7 | 0.153 |
| Transcription | *CEBPA* | 19 | 21.6 | 238 | 15.6 | 0.237 |
|  | *CEBPA,* biallelic | 4 | 4.5 | 88 | 5.8 | 1.000 |
|  | *CEBPA-*TAD | 4 | 4.5 | 33 | 2.2 | 0.096 |
|  | *CEBPA-*bZIP (in frame) | 5 | 5.7 | 139 | 9.1 | **0.004** |
|  | *CUX1* | 6 | 6.8 | 36 | 2.4 | **0.024** |
|  | *GATA2* | 4 | 4.5 | 92 | 6.0 | 0.816 |
|  | *IKZF1* | 0 | 0.0 | 45 | 2.9 | 0.172 |
|  | *PHF6* | 4 | 4.5 | 48 | 3.1 | 0.527 |
|  | *RUNX1* | 16 | 18.2 | 135 | 8.8 | **0.007** |
|  | *WT1* | 2 | 2.3 | 116 | 7.6 | 0.059 |
|  | *ETV6* | 0 | 0.0 | 15 | 1.0 | 1.000 |
| Signaling | *FLT3-*ITD | 8 | 9.1 | 340 | 22.3 | **0.002** |
|  | *FLT3-*TKD | 5 | 5.7 | 64 | 4.2 | 1.000 |
|  | *NRAS* | 12 | 13.6 | 237 | 15.5 | 0.762 |
|  | *KRAS* | 2 | 2.3 | 83 | 5.4 | 0.321 |
|  | *KIT* | 2 | 2.3 | 77 | 5.0 | 0.315 |
|  | *NOTCH1* | 1 | 1.1 | 31 | 2.0 | 1.000 |
|  | *CSF3R* | 1 | 1.1 | 28 | 1.8 | 1.000 |
|  | *CBL* | 2 | 2.3 | 30 | 2.0 | 0.694 |
|  | *PTPN11* | 4 | 4.5 | 109 | 7.1 | 0.517 |
| Splicing | *SF3B1* | 3 | 3.4 | 43 | 2.8 | 0.737 |
|  | *SRSF2* | 24 | 27.3 | 78 | 5.1 | **<0.001** |
|  | *U2AF1* | 3 | 3.4 | 42 | 2.8 | 0.734 |
|  | *ZRSR2* | 4 | 4.5 | 21 | 1.4 | **0.044** |
| other | *TP53* | 3 | 3.4 | 112 | 7.3 | 0.202 |
|  | *NPM1* | 13 | 14.8 | 488 | 32.0 | **<0.001** |
| Cohesin | *RAD21* | 0 | 0.0 | 51 | 3.3 | 0.110 |
|  | *SMC1A* | 0 | 0.0 | 25 | 1.6 | 0.633 |
|  | *SMC3* | 1 | 1.1 | 19 | 1.2 | 0.619 |
| cytogenetics | normal karyotype | 66 | 75.0 | 769 | 50.4 | **<0.001** |
|  | komplex karyotype | 5 | 5.7 | 183 | 12.0 | 0.196 |
|  | t(8;21)(q22;q22.1) | 0 | 0.0 | 61 | 4.0 | 0.107 |
|  | inv(16)(p13.1q22) or t(16;16)(p13.1;q22) | 0 | 0.0 | 58 | 3.8 | 0.168 |

**Table S5 Co-mutational pattern of AML with mutated *STAG2*.** Abbreviations: absolute (abs.), number (n/N), relative (rel.). Bold typing indicates statistical significance (*p*<0.05).

| **Variable name** | **Odds ratio [95%-CI]** | ***p*** |
| --- | --- | --- |
| mutated *STAG2* | 0.97 [0.60-1.57] | 0.904 |
| mutated *BCOR* | 0.88 [0.53-1.46] | 0.627 |
| mutated *RUNX1* | 0.36 [0.25-0.51] | **<0.001** |
| mutated *ASXL1* | 0.46 [0.32-0.67] | **<0.001** |

**Table S6** Multivariable analysis regarding the impact of *STAG2* mutations status with respect to achievement of complete remission after intensive induction therapy adjusted for frequent co-mutations in *BCOR*, *RUNX1*, and *ASXL1*

| **Variable name** | **Hazard ratio [95%-CI]** | ***p*** |
| --- | --- | --- |
| mutated *STAG2* | 0.88 [0.69-1.14] | 0.354 |
| mutated *BCOR* | 1.39 [1.08-1.79] | **0.009** |
| mutated *RUNX1* | 1.63 [1.36-1.96] | **<0.001** |
| mutated *ASXL1* | 1.56 [1.27-1.91] | **<0.001** |

**Table S7** Multivariable analysis regarding the impact of *STAG2* mutations status with respect to event-free survival adjusted for frequent co-mutations in *BCOR*, *RUNX1*, and *ASXL1*

| **Variable name** | **Hazard ratio [95%-CI]** | ***p*** |
| --- | --- | --- |
| mutated *STAG2* | 0.70 [0.48-1.00] | 0.052 |
| mutated *BCOR* | 1.38 [0.99-1.92] | 0.058 |
| mutated *RUNX1* | 1.58 [1.20-2.09] | **0.001** |
| mutated *ASXL1* | 1.28 [0.95-1.73] | 0.107 |

**Table S8** Multivariable analysis regarding the impact of *STAG2* mutations status with respect to relapse-free survival adjusted for frequent co-mutations in *BCOR*, *RUNX1*, and *ASXL1*

| **Variable name** | **Odds ratio [95%-CI]** | ***p*** |
| --- | --- | --- |
| mutated *STAG2* | 0.80 [0.61-1.06] | 0.115 |
| mutated *BCOR* | 1.20 [0.92-1.57] | 0.186 |
| mutated *RUNX1* | 1.64 [1.36-1.99] | **<0.001** |
| mutated *ASXL1* | 1.47 [1.19-1.80] | **<0.001** |

**Table S9** Multivariable analysis regarding the impact of *STAG2* mutations status with respect to overall survival adjusted for frequent co-mutations in *BCOR*, *RUNX1*, and *ASXL1*

| **Parameter** | ***RAD21* mutated** | ***RAD21* wildtype** | ***p*** |
| --- | --- | --- | --- |
| **n/N (%)** | 51/1615 (3.2) | 1564/1615 (96.8) |  |
| **Age (years), median (IQR)** | 55 (47-60) | 56 (44-66) | 0.666 |
| **Sex, n (%)** |  |  | 0.321 |
| female | 28 (54.9) | 740 (47.6) |  |
| male | 23 (45.1) | 815 (52.4) |  |
| **Disease status, n (%)** |  |  |  |
| de novo | 44 (88.0) | 1295 (84.2) | 0.559 |
| sAML | 5 (10.0) | 190 (12.3) | 0.826 |
| tAML | 1 (2.0) | 53 (3.5) | 1.000 |
| **extramedullary disease, n (%)** | 9 (17.6) | 205 (13.1) | 0.285 |
| **ELN-Risk 2022, n (%)** |  |  |  |
| favorable | 26 (51.0) | 551 (35.2) | **0.037** |
| intermediate | 16 (31.3) | 408 (26.1) | 0.425 |
| adverse | 8 (15.7) | 578 (37.0) | **0.001** |
| missing | 1 (2.0) | 27 (1.7) |  |
| **Complex karyotype, n (%)** |  |  | **0.015** |
| No | 47 (97.9) | 1220 (86.7) |  |
| Yes | 1 (2.1) | 187 (13.3) |  |
| **Normal karyotype, n (%)** |  |  | **0.009** |
| No | 13 (26.5) | 660 (45.4) |  |
| Yes | 36 (73.5) | 794 (54.6) |  |
| **allogeneic HCT** |  |  |  |
| in first CR | 5 (9.8) | 239 (15.3) | 0.327 |
| overall | 10 (19.6) | 509 (32.5) | **0.049** |
| **Laboratory, median (IQR)** |  |  |  |
| WBC (10^9^/l) | 38.1 (6.5-88.7) | 18.8 (4.4-52.9) | 0.062 |
| HB (mmol/l) | 5.8 (4.8-6.6) | 5.9 (5.1-7.0) | 0.217 |
| PLT (10^9^/l) | 52.0 (28.0-88.0) | 50.0 (27.0-95.0) | 0.739 |
| LDH (U/l) | 705.0 (472.0-1382.0) | 440.2 (278.0-756.6) | **<0.001** |
| PBB (%) | 39.0 (10.5-66.5) | 40.0 (12.0-73.0) | 0.767 |
| BMB (%) | 54.5 (42.0-79.5) | 63.0 (44.3-79.0) | 0.453 |

**Table S10 Baseline patient characteristics with respect to *RAD21* mutation status.**Abbreviations: acute myeloid leukemia (AML), secondary AML (sAML), therapy-associated AML (tAML), bone marrow blasts (BMB), complete remission (CR), hemoglobin (HB), hematopoietic cell transplantation (HCT), interquartile range (IQR), number (n/N), peripheral blood blasts (PBB), platelet count (PLT), white blood cell count (WBC). Boldface indicates statistical significance (*p*<0.05).

|  |  | ***RAD21* mut. (abs)** | ***RAD21* mut. (rel) %** | ***RAD21* wt. (abs.)** | ***RAD21* wt (rel) %** | ***p*** |
| --- | --- | --- | --- | --- | --- | --- |
|  | N=1615 | 51 | 3.2 | 1564 | 96.3 |  |
| Epigenetic | *DNMT3A* | 15 | 29.4 | 441 | 28.2 | 0.875 |
|  | *IDH1* | 2 | 3.9 | 147 | 9.4 | 0.225 |
|  | *IDH2* | 6 | 11.8 | 222 | 14.2 | 0.838 |
|  | *TET2* | 10 | 19.6 | 300 | 19.2 | 1.000 |
|  | *BCOR* | 0 | 0.0 | 76 | 4.9 | 0.170 |
|  | *BCORL1* | 0 | 0.0 | 60 | 3.8 | 0.258 |
|  | *ASXL1* | 2 | 3.9 | 132 | 8.4 | 0.434 |
|  | *EZH2* | 5 | 9.8 | 58 | 3.7 | **0.046** |
| Transcription | *CEBPA* | 12 | 23.5 | 246 | 15.7 | 0.182 |
|  | *CEBPA,* biallelic | 6 | 11.8 | 86 | 5.5 | 0.119 |
|  | *CEBPA-*TAD | 0 | 0.0 | 37 | 2.4 | 0.639 |
|  | *CEBPA-*bZIP (in frame) | 7 | 13.7 | 137 | 8.8 | 0.721 |
|  | *CUX1* | 0 | 0.0 | 42 | 2.7 | 0.641 |
|  | *GATA2* | 5 | 9.8 | 92 | 5.9 | 0.230 |
|  | *IKZF1* | 0 | 0.0 | 45 | 2.9 | 0.398 |
|  | *PHF6* | 1 | 2.0 | 52 | 3.3 | 1.000 |
|  | *RUNX1* | 1 | 2.0 | 150 | 9.6 | 0.082 |
|  | *WT1* | 5 | 9.8 | 113 | 7.2 | 0.417 |
|  | *ETV6* | 0 | 0.0 | 15 | 1.0 | 1.000 |
| Signaling | *FLT3-ITD* | 15 | 29.4 | 333 | 21.3 | 0.227 |
|  | *FLT3-TKD* | 5 | 9.8 | 64 | 4.1 | 0.232 |
|  | *NRAS* | 11 | 21.6 | 239 | 15.3 | 0.237 |
|  | *KRAS* | 7 | 13.7 | 78 | 5.0 | **0.016** |
|  | *KIT* | 2 | 3.9 | 77 | 4.9 | 1.000 |
|  | *NOTCH1* | 1 | 2.0 | 31 | 2.0 | 1.000 |
|  | *CSF3R* | 1 | 2.0 | 28 | 1.8 | 0.610 |
|  | *CBL* | 4 | 7.8 | 28 | 1.8 | **0.017** |
|  | *PTPN11* | 6 | 11.8 | 109 | 7.0 | 0.173 |
| Splicing | *SF3B1* | 1 | 2.0 | 45 | 2.9 | 1.000 |
|  | *SRSF2* | 2 | 3.9 | 100 | 6.4 | 0.768 |
|  | *U2AF1* | 0 | 0.0 | 45 | 2.9 | 0.398 |
|  | *ZRSR2* | 0 | 0.0 | 25 | 1.6 | 1.000 |
| other | *TP53* | 0 | 0.0 | 115 | 7.4 | **0.047** |
|  | *NPM1* | 29 | 56.9 | 475 | 30.4 | **<0.001** |
| Cohesin | *STAG2* | 0 | 0.0 | 88 | 5.6 | 0.110 |
|  | *SMC1A* | 0 | 0.0 | 25 | 1.6 | 1.000 |
|  | *SMC3* | 0 | 0.0 | 20 | 1.3 | 1.000 |
| cytogenetics | normal karyotype | 36 | 70.6 | 794 | 50.8 | **0.009** |
|  | komplex karyotype | 1 | 2.0 | 187 | 12.0 | **0.015** |
|  | t(8;21)(q22;q22.1) | 4 | 7.8 | 57 | 3.6 | 0.151 |
|  | inv(16)(p13.1q22) or t(16;16)(p13.1;q22) | 1 | 2.0 | 57 | 3.6 | 0.719 |

**Table S11 Co-mutational pattern of AML with mutated *RAD21*.** Abbreviations: absolute (abs.), number (n/N), relative (rel.). Bold typing indicates statistical significance (*p*<0.05).

| **Parameter** | ***SMC1A* mutated** | ***SMC1A* wildtype** | ***p*** |
| --- | --- | --- | --- |
| **n/N (%)** | 25/1615 (1.5) | 1590/1615 (98.5) |  |
| **Age (years), median (IQR)** | 53 (48.0-66.0) | 56 (44.0-65.0) | 0.965 |
| **Sex, n (%)** |  |  | 0.546 |
| female | 10 (40.0) | 760 (48.0) |  |
| male | 15 (60.0) | 823 (52.0) |  |
| **Disease status, n (%)** |  |  |  |
| de novo | 20 (87.0) | 1319 (84.3) | 1.000 |
| sAML | 2 (8.7) | 193 (12.3) | 1.000 |
| tAML | 1 (4.3) | 53 (3.4) | 0.548 |
| **extramedullary disease, n (%)** | 2 (8.0) | 212 (13.3) | 0.759 |
| **ELN-Risk 2022, n (%)** |  |  |  |
| favorable | 13 (52.0) | 564 (35.5) | 0.050 |
| intermediate | 6 (24.0) | 418 (26.3) | 1.000 |
| adverse | 4 (16.0) | 582 (36.6) | 0.053 |
| missing | 2 (8.0) | 26 (1.6) |  |
| **Complex karyotype, n (%)** |  |  | 1.000 |
| No | 20 (87.0) | 1247 (87.1) |  |
| Yes | 3 (13.0) | 185 (12.9) |  |
| **Normal karyotype, n (%)** |  |  | 1.000 |
| No | 10 (45.5) | 663 (44.8) |  |
| Yes | 12 (54.5) | 818 (55.2) |  |
| **allogeneic HCT** |  |  |  |
| in first CR | 3 (12.0) | 241 (15.2) | 1.000 |
| overall | 3 (12.0) | 516 (32.5) | 0.069 |
| **Laboratory, median (IQR)** |  |  |  |
| WBC (10^9^/l) | 16.7 (3.1-44.9) | 19.1 (4.6-53.6) | 0.415 |
| HB (mmol/l) | 5.9 (4.2-6.5) | 5.9 (5.1-7.0) | 0.172 |
| PLT (10^9^/l) | 38 (12.5-72.0) | 50.0 (27.0-95.0) | 0.237 |
| LDH (U/l) | 503.0 (300.0-1141.0) | 443.7 (281.0-775.0) | 0.456 |
| PBB (%) | 50.0 (20.0-74.0) | 40.0 (12.0-73.0) | 0.349 |
| BMB (%) | 67.0 (52.0-83.5) | 62.8 (44.0-79.0) | 0.307 |

**Table S12 Baseline patient characteristics with respect to *SMC1A* mutation status.**Abbreviations: acute myeloid leukemia (AML), secondary AML (sAML), therapy-associated AML (tAML), bone marrow blasts (BMB), complete remission (CR), hemoglobin (HB), hematopoietic cell transplantation (HCT), interquartile range (IQR), number (n/N), peripheral blood blasts (PBB), platelet count (PLT), white blood cell count (WBC). Boldface indicates statistical significance (*p*<0.05).

| **Parameter** | ***SMC3* mutated** | ***SMC3* wildtype** | ***p*** |
| --- | --- | --- | --- |
| **n/N (%)** | 20/1615 (1.2) | 1595/1615 (98.8) |  |
| **Age (years), median (IQR)** | 55.5 (47.0-67.0) | 56.0 (44.0-65.0) | 0.616 |
| **Sex, n (%)** |  |  | 0.654 |
| female | 11 (0.55) | 761 (47.9) |  |
| male | 9 (0.45) | 829 (52.1) |  |
| **Disease status, n (%)** |  |  |  |
| de novo | 16 (88.8) | 1323 (84.3) | 0.753 |
| sAML | 1 (5.6) | 194 (12.4) | 0.714 |
| tAML | 1 (5.6) | 53 (3.3) | 0.462 |
| **extramedullary disease, n (%)** | 5 (25.0) | 209 (13.1) | 0.109 |
| **ELN-Risk 2022, n (%)** |  |  |  |
| favorable | 8 (40.0) | 559 (35.0) | 0.470 |
| intermediate | 4 (20.0) | 420 (26.3) | 0.794 |
| adverse | 6 (30.0) | 580 (36.4) | 0.812 |
| missing | 2 (10.0) | 36 (2.3) |  |
| **Complex karyotype, n (%)** |  |  | 1.000 |
| No | 14 (87.5) | 1253 (87.1) |  |
| Yes | 2 (12.5) | 186 (12.9) |  |
| **Normal karyotype, n (%)** |  |  | 0.059 |
| No | 4 (22.2) | 669 (45.1) |  |
| Yes | 14 (77.8) | 816 (54.9) |  |
| **allogeneic HCT** |  |  |  |
| in first CR | 1 (5.0) | 243 (15.2) | 0.503 |
| overall | 5 (25.0) | 514 (32.2) | 0.803 |
| **Laboratory, median (IQR)** |  |  |  |
| WBC (10^9^/l) | 34.0 (5.2-74.4) | 19.0 (4.4-53.0) | 0.279 |
| HB (mmol/l) | 6.1 (5.5-6.6) | 5.9 (5.0-7.0) | 0.332 |
| PLT (10^9^/l) | 39.0 (30.0-93.0) | 50.0 (27.0-94.5) | 0.996 |
| LDH (U/l) | 556.0 (326.0-817.0) | 440.0 (280.0-775.0) | 0.452 |
| PBB (%) | 46.0 (15.0-76.0) | 40.0 (12.0-73.0) | 0.733 |
| BMB (%) | 71.0 (49.5-81.0) | 63.0 (44.0-79.0) | 0.400 |

**Table S13 Baseline patient characteristics with respect to *SMC3* mutation status.**Abbreviations: acute myeloid leukemia (AML), secondary AML (sAML), therapy-associated AML (tAML), bone marrow blasts (BMB), complete remission (CR), hemoglobin (HB), hematopoietic cell transplantation (HCT), interquartile range (IQR), number (n/N), peripheral blood blasts (PBB), platelet count (PLT), white blood cell count (WBC). Boldface indicates statistical significance (*p*<0.05).

|  |  | ***SMC1A* mut. (abs)** | ***SMC1A* mut. (rel) %** | ***SMC1A* wt. (abs)** | ***SMC1A* wt (rel) %** | ***p*** |
| --- | --- | --- | --- | --- | --- | --- |
|  | N=1615 | 25 | 1.5 | 1590 | 98.4 |  |
| Epigenetic | *DNMT3A* | 8 | 32.0 | 448 | 28.2 | 0.659 |
|  | *IDH1* | 2 | 8.0 | 147 | 9.2 | 1.000 |
|  | *IDH2* | 4 | 16.0 | 224 | 14.1 | 0.772 |
|  | *TET2* | 7 | 28.0 | 303 | 19.1 | 0.303 |
|  | *BCOR* | 0 | 0.0 | 76 | 4.8 | 0.628 |
|  | *BCORL1* | 0 | 0.0 | 60 | 3.8 | 1.000 |
|  | *ASXL1* | 2 | 8.0 | 132 | 8.3 | 1.000 |
|  | *EZH2* | 0 | 0.0 | 63 | 4.0 | 0.622 |
| Transcription | *CEBPA* | 6 | 24.0 | 252 | 15.8 | 0.287 |
|  | *CEBPA,* biallelic | 1 | 4.0 | 91 | 5.7 | 1.000 |
|  | *CEBPA-*TAD | 2 | 8.0 | 35 | 2.2 | 0.128 |
|  | *CEBPA-*bZIP (in frame) | 2 | 8.0 | 142 | 8.9 | 0.340 |
|  | *CUX1* | 0 | 0.0 | 42 | 2.6 | 1.000 |
|  | *GATA2* | 0 | 0.0 | 97 | 6.1 | 0.397 |
|  | *IKZF1* | 0 | 0.0 | 45 | 2.8 | 1.000 |
|  | *PHF6* | 0 | 0.0 | 52 | 3.3 | 1.000 |
|  | *RUNX1* | 0 | 0.0 | 151 | 9.5 | 0.161 |
|  | *WT1* | 1 | 4.0 | 117 | 7.4 | 1.000 |
|  | *ETV6* | 0 | 0.0 | 15 | 0.9 | 1.000 |
| Signaling | *FLT3-*ITD | 5 | 20.0 | 343 | 21.6 | 1.000 |
|  | *FLT3-*TKD | 3 | 12.0 | 66 | 4.2 | 0.191 |
|  | *NRAS* | 3 | 12.0 | 247 | 15.5 | 0.786 |
|  | *KRAS* | 3 | 12.0 | 82 | 5.2 | 0.142 |
|  | *KIT* | 1 | 4.0 | 78 | 4.9 | 1.000 |
|  | *NOTCH1* | 1 | 4.0 | 31 | 1.9 | 0.397 |
|  | *CSF3R* | 1 | 4.0 | 28 | 1.8 | 0.367 |
|  | *CBL* | 0 | 0.0 | 32 | 2.0 | 1.000 |
|  | *PTPN11* | 2 | 8.0 | 113 | 7.1 | 0.698 |
| Splicing | *SF3B1* | 0 | 0.0 | 46 | 2.9 | 1.000 |
|  | *SRSF2* | 2 | 8.0 | 100 | 6.3 | 0.670 |
|  | *U2AF1* | 0 | 0.0 | 45 | 2.8 | 1.000 |
|  | *ZRSR2* | 1 | 4.0 | 24 | 1.5 | 0.326 |
| other | *TP53* | 1 | 4.0 | 114 | 7.2 | 1.000 |
|  | *NPM1* | 10 | 40.0 | 494 | 31.1 | 0.391 |
| Cohesin | *RAD21* | **0** | 0.0 | 51 | 3.2 | 1.000 |
|  | *STAG2* | **0** | 0.0 | 88 | 5.5 | 0.623 |
|  | *SMC3* | **0** | 0.0 | 20 | 1.3 | 1.000 |
| cytogenetics | normal karyotype | 12 | 48.0 | 818 | 51.4 | 1.000 |
|  | komplex karyotype | 3 | 12.0 | 185 | 11.6 | 1.000 |
|  | t(8;21)(q22;q22.1) | 5 | 20.0 | 56 | 3.5 | **0.002** |
|  | inv(16)(p13.1q22) or t(16;16)(p13.1;q22) | 0 | 0.0 | 58 | 3.6 | 0.620 |

**Table S14 Co-mutational pattern of AML with mutated *SMC1A*.** Abbreviations: absolute (abs.), number (n/N), relative (rel.). Bold typing indicates statistical significance (*p*<0.05).

|  |  | ***SMC3* mut. (abs)** | ***SMC3* mut. (rel) %** | ***SMC3* wt. (abs)** | ***SMC3* wt. (rel) %** | **p** |
| --- | --- | --- | --- | --- | --- | --- |
|  | N=1615 | 20 | 1.2 | 1595 | 98.2 |  |
| Epigenetic | *DNMT3A* | 8 | 40.0 | 448 | 28.1 | 0.316 |
|  | *IDH1* | 2 | 10.0 | 147 | 9.2 | 0.707 |
|  | *IDH2* | 3 | 15.0 | 225 | 14.1 | 0.755 |
|  | *TET2* | 5 | 25.0 | 305 | 19.1 | 0.566 |
|  | *BCOR* | 1 | 5.0 | 75 | 4.7 | 1.000 |
|  | *BCORL1* | 1 | 5.0 | 59 | 3.7 | 0.534 |
|  | *ASXL1* | 1 | 5.0 | 133 | 8.3 | 1.000 |
|  | *EZH2* | 0 | 0.0 | 63 | 3.9 | 1.000 |
| Transcription | *CEBPA* | 2 | 10.0 | 256 | 16.1 | 0.557 |
|  | *CEBPA,* biallelic | 0 | 0.0 | 92 | 5.8 | 0.623 |
|  | *CEBPA-*TAD | 2 | 10.0 | 35 | 2.2 | 0.900 |
|  | *CEBPA-*bZIP (in frame) | 0 | 0.0 | 144 | 9.0 | 0.114 |
|  | *CUX1* | 0 | 0.0 | 42 | 2.6 | 1.000 |
|  | *GATA2* | 1 | 5.0 | 96 | 6.0 | 1.000 |
|  | *IKZF1* | 0 | 0.0 | 45 | 2.8 | 1.000 |
|  | *PHF6* | 0 | 0.0 | 53 | 3.3 | 1.000 |
|  | *RUNX1* | 3 | 15.0 | 148 | 9.3 | 0.425 |
|  | *WT1* | 0 | 0.0 | 118 | 7.4 | 0.393 |
|  | *ETV6* | 0 | 0.0 | 15 | 0.9 | 1.000 |
| Signaling | *FLT3-*ITD | 6 | 30.0 | 342 | 21.4 | 0.414 |
|  | *FLT3-*TKD | 2 | 10.0 | 67 | 4.2 | 0.347 |
|  | *NRAS* | 6 | 30.0 | 244 | 15.3 | 0.110 |
|  | *KRAS* | 0 | 0.0 | 85 | 5.3 | 0.621 |
|  | *KIT* | 0 | 0.0 | 79 | 5.0 | 0.620 |
|  | *NOTCH1* | 0 | 0.0 | 32 | 2.0 | 1.000 |
|  | *CSF3R* | 0 | 0.0 | 29 | 1.8 | 1.000 |
|  | *CBL* | 1 | 5.0 | 31 | 1.9 | 0.332 |
|  | *PTPN11* | 3 | 15.0 | 112 | 7.0 | 0.167 |
| Splicing | *SF3B1* | 0 | 0.0 | 46 | 2.9 | 1.000 |
|  | *SRSF2* | 3 | 15.0 | 99 | 6.2 | 0.129 |
|  | *U2AF1* | 0 | 0.0 | 45 | 2.8 | 1.000 |
|  | *ZRSR2* | 0 | 0.0 | 25 | 1.6 | 1.000 |
| other | *TP53* | 1 | 5.0 | 114 | 7.1 | 1.000 |
|  | *NPM1* | 13 | 65.0 | 491 | 30.8 | **0.003** |
| Cohesin | *RAD21* | 0 | 0.0 | 51 | 3.2 | 1.000 |
|  | *SMC1A* | 0 | 0.0 | 25 | 1.6 | 1.000 |
|  | *STAG2* | 0 | 0.0 | 88 | 5.5 | 0.619 |
| cytogenetics | normal karyotype | 14 | 70.0 | 816 | 51.2 | 0.059 |
|  | komplex karyotype | 2 | 10.0 | 186 | 11.7 | 1.000 |
|  | t(8;21)(q22;q22.1) | 1 | 5.0 | 60 | 3.8 | 0.525 |
|  | inv(16)(p13.1q22) or t(16;16)(p13.1;q22) | 0 | 0.0 | 58 | 3.6 | 1.000 |

**Table S15 Co-mutational pattern of AML with mutated *SMC3*.** Abbreviations: absolute (abs.), number (n/N), relative (rel.). Bold typing indicates statistical significance (*p*<0.05).
